# THE IMPACT OF OBESITY ON THE CLINICAL COURSE OF INFLAMMATORY BOWEL DISEASE

**DOI:** 10.1590/S0004-2803.24612025-093

**Published:** 2026-05-25

**Authors:** Roberta Oliveira Raimundo BORSATO, Júlio Maria Fonseca CHEBLI, Tarsila Campanha da Rocha RIBEIRO, Luiz Henrique Silva BORSATO, Marcos Paulo Moraes SALES, Maria Antônia de Lima BARRA

**Affiliations:** * Universidade Federal de Juiz de Fora, Faculdade de Medicina, Juiz de Fora, Brasil.

**Keywords:** Inflammatory bowel disease, Crohn’s disease, ulcerative colitis, obesity, metabolic syndrome, Doença inflamatória intestinal, doença de crohn, retocolite ulcerativa, obesidade, síndrome metabólica

## Abstract

**Background:**

The incidence and prevalence of disorders such as obesity, metabolic syndrome (MS), and inflammatory bowel disease (IBD) have increased over recent decades. MS is a complex condition represented by a cluster of cardiovascular risk factors with a multifactorial origin-including genetic, behavioral, dietary factors, and alterations in gut microbiota. IBD reflects a complex and heterogeneous immune-mediated condition that typically, though not exclusively, affects the intestine.

**Objective:**

To evaluate the impact of obesity on the clinical course of IBD in a cohort of patients followed at a referral center for IBD.

**Methods:**

This was a retrospective longitudinal observational cohort study including patients of both sexes and all ethnicities, followed at the Inflammatory Bowel Disease Referral Center of the University Hospital of the Federal University of Juiz de Fora, between January 2019 and August 2023. A total of 404 adults aged 18 to 80 years with a diagnosis of IBD-established by clinical, endoscopic/histological, and/or imaging criteria-were included. IBD cases were classified as either Crohn’s disease (CD) or ulcerative colitis (UC). To assess DII annual activity, electronic medical records were reviewed for outpatient visits and hospitalizations from 2019 to 2023. CD was considered active when the Harvey-Bradshaw Index (HBI) was ≥5, and UC was considered active when the total Mayo score was ≥3 or the partial Mayo score was ≥2.

**Results:**

The mean age at IBD diagnosis was 37.79±13.44 years, with an average disease duration of 12.50±8.3 years. Biologic therapy was used in the majority of patients (58.4%), with treatment failure occurring in 56.8% of these cases during the study period. No statistically significant differences were observed in most outcomes, except for a higher frequency of biologic use in non-obese patients with CD (*P*=0.028). There was a trend toward a greater number of years with active disease in obese patients with CD (*P*=0.062).

**Conclusion:**

IBD patients with obesity were predominantly female. Among those with CD, the phenotype was mainly non-stricturing, non-penetrating, with a clinical course characterized by greater disease activity over time, suggesting that obesity may be an unfavorable factor for disease control. This trend was not observed among patients with UC.

## HIGHLIGHTS

• To evaluate the impact of obesity on the clinical course of inflammatory bowel disease (IBD) in a cohort of patients followed at an IBD referral center.

• Retrospective longitudinal observational cohort study including 404 patients diagnosed with IBD, classified as having either Crohn’s disease (CD) or ulcerative colitis (UC).

• No statistically significant differences were observed in most of the outcomes analyzed, except for a higher frequency of biologic therapy use among non-obese patients with CD. There was also a trend toward a greater number of years with active disease in obese patients with CD.

• IBD patients with obesity were predominantly female. Among those with CD, the disease phenotype was primarily inflammatory, with a clinical course marked by greater disease activity over time, suggesting that obesity may be an unfavorable factor for disease control. This trend was not observed in patients with UC.

## RESUMO

**Contexto:**

A incidência e prevalência de desordens como obesidade, síndrome metabólica (SM) e doença inflamatória intestinal (DII) aumentaram nas últimas décadas. A SM é um transtorno complexo representado por um conjunto de fatores de risco cardiovascular, tendo origem multifatorial: envolve fatores genéticos, padrões comportamentais e dietéticos, e alterações na microbiota intestinal. A DII reflete uma condição imuno mediada complexa e heterogênea que tipicamente, mas não exclusivamente, afeta o intestino.

**Objetivo:**

Avaliar o impacto da obesidade no curso clínico da DII em uma coorte de pacientes acompanhados em um centro de referência em DII.

**Métodos:**

Trata-se de estudo de coorte observacional longitudinal retrospectivo, que incluiu pacientes de ambos os sexos e de qualquer etnia, acompanhados no Centro de Referência em Doenças Inflamatórias Intestinais do Hospital Universitário da Universidade Federal de Juiz de Fora (HU-UFJF), no período compreendido entre janeiro de 2019 a agosto de 2023.Foram incluídos 404 adultos entre 18 e 80 anos, utilizando-se de alguns critérios de exclusão, que possuíam o diagnóstico de DII, estabelecido por critérios clínicos, endoscópicos/histológicos e/ou de imagem. Quanto ao tipo de DII, foi dividido em DC ou RCU. Para avaliação da atividade da doença, foram analisados os registros eletrônicos de cada paciente durante suas consultas ambulatoriais ou hospitalizações entre os anos de 2019 e 2023, sendo que, para DC, foi considerado como atividade de doença HBI ≥5, e para RCU, quando classificado pelo Mayo total, foi considerada atividade de doença quando Mayo total ≥3, e quando analisado pelo Mayo parcial, atividade com Mayo parcial ≥2.

**Resultados:**

A média de idade ao diagnóstico de DII foi de 37,79±13,44 anos e média de duração da doença de 12,50±8,3 anos. A terapia biológica foi empregada na maioria dos pacientes (58,4%), sendo que houve falha da terapia em 56,8% desses pacientes durante o período analisado. Não foi observada diferença estatisticamente significante nos diversos desfechos analisados, exceto no uso de biológicos mais frequente em pacientes com DC não obesos (*P*=0,028). Houve uma tendência para maior número de anos com atividade da doença em pacientes obesos com DC (*P*=0.062).

**Conclusão:**

Foi possível concluir que pacientes com DII e obesidade eram predominantemente mulheres. Aqueles com DC apresentaram fenótipo principalmente inflamatório e curso evolutivo caracterizado por maior atividade de doença ao longo do seguimento, sugerindo que a obesidade pode ser um fator desfavorável para o controle da DC. A mesma tendência não foi observada entre os pacientes com RCU.

## INTRODUCTION

The incidence and prevalence of disorders such as obesity, metabolic syndrome (MS), and inflammatory bowel disease (IBD) have increased significantly in recent decades. At the turn of the 21st century, IBD became a global disease, with rising incidence in industrialized countries where a westernization of societies was observed^1^. Among individuals with IBD, the rate of obesity also appears to be increasing, both in adult and pediatric populations^2^ ^,^ ^3^. Several factors may contribute to the development of IBD, including obesity, physical inactivity, smoking, a diet rich in processed foods, sugars, and saturated fats, antibiotic use, alterations in the gut microbiota, excessive hygiene, and genetic predisposition^3^ ^,^ ^4^. It is plausible that a diet rich in processed foods and macronutrients such as simple sugars, saturated fats, and omega-6 fatty acids, along with additives such as colorings and emulsifiers, induces intestinal dysbiosis, which in turn promotes local inflammation^4^.

IBD is a chronic, immune-mediated, and heterogeneous inflammatory condition that typically-but not exclusively-affects the intestines. In turn, it was observed that in patients with central obesity, the expansion of adipocytes leads to local hypoxia and infiltration of adipose tissue by immune cells. This infiltration triggers the release of pro-inflammatory cytokines such as tumor necrosis factor-alpha (TNFα) and interleukin-6 (IL-6), as well as the suppression of the anti-inflammatory adiponectin, ultimately resulting in metabolic dysfunction and insulin resistance^4^ ^,^ ^5^. Alterations in the gut microbiota may compromise the intestinal mucosal barrier, leading to bacterial and metabolite translocation, thereby amplifying systemic inflammation and contributing to metabolic diseases^6^.

The relationship between IBD and metabolic disorders has been reported in several studies. One such example is the interaction between IBD and metabolic dysfunction-associated steatotic liver disease (MASLD), where eutrophic patients with IBD showed higher rates of MASLD compared to eutrophic individuals without IBD^7^. Additionally, it has been shown that 42% of patients with IBD have MASLD, with 9.5% of them presenting with advanced liver fibrosis. Moreover, IBD appears to be an independent risk factor for hepatic fibrosis, suggesting that chronic intestinal inflammation may contribute to liver disease^5^. On the other hand, how obesity impacts the pharmacological management of IBD remains uncertain^2^ ^-^ ^8^. While some data suggest that pharmacokinetic mechanisms in obese patients may reduce the response to IBD-targeted therapy, the real clinical impact of these mechanisms on the response to various therapeutic agents used in IBD remains unclear and requires more refined studies^2^. Obesity may influence the absorption, distribution, and metabolism of biological agents, but few pharmacokinetic studies provide in-depth data in this field. Interestingly, one study showed that even a 5% reduction in baseline body weight can lead to better outcomes in the treatment and progression of immune-mediated inflammatory diseases^9^. However, dietary and lifestyle interventions are challenging, especially for patients with IBD. Thus, weight-loss medications or even bariatric surgery may offer a more effective alternative, leading to significant weight loss in a larger proportion of patients^9^.

To date, no studies in Brazil have evaluated the impact of obesity on the phenotype and long-term clinical course of IBD, particularly whether obesity affects patient management, treatment response, or increases the risk of IBD-related complications and the need for intestinal resections. Therefore, the aim of the present study is to assess the impact of obesity on the clinical course of IBD in a cohort of patients followed at a tertiary referral center for IBD.

## METHODS

The study was approved by the Research Ethics Committee of the University Hospital of the Federal University of Juiz de Fora (CEP-HU-UFJF) under protocol number 5.889.145. Informed consent was waived, as this is a retrospective study using secondary data obtained from medical records of patients followed at the IBD Center of HU-UFJF.

This is a retrospective longitudinal observational cohort study that included patients of both sexes and any ethnicity, followed at the Reference Center for Inflammatory Bowel Diseases at the University Hospital of the Federal University of Juiz de Fora (HU-UFJF), from January 2019 to August 2023. Patients under 18 or over 80 years of age, pregnant or lactating women during the study period, malnourished individuals (defined as having a BMI <18.5 kg/m²), and those with incomplete medical records were excluded.

The diagnosis of IBD was based on clinical, endoscopic/histological, and/or imaging criteria well established. Data were extracted from both electronic and physical medical records, including sociodemographic and clinical characteristics (age, sex, ethnicity, height, current weight, BMI, former or current smoking status, associated chronic diseases such as systemic arterial hypertension, diabetes mellitus, dyslipidemia, metabolic syndrome, hepatic steatosis detected by ultrasound), and IBD-related characteristics, including IBD type, disease phenotype and location, age at diagnosis, disease duration until study inclusion, current and previous treatments, annual disease activity, presence of extraintestinal manifestations, history of IBD-related surgery and its possible complications, history of severe or opportunistic infections, and hospitalizations due to IBD complications. Severe infection was defined as one requiring intravenous antibiotic therapy or hospitalization.

For each patient using biologic therapy, we evaluated treatment failure and the need to switch medications from 2019 until the end of the study, as well as the need for hospitalization due to IBD complications, occurrence of opportunistic infections, and the need for surgical intervention and its possible complications.

IBD type was classified as either crohn’s disease (CD) or ulcerative colitis (UC), and disease phenotype and location were determined using the Montreal classification^10^. For CD, we classified patients into non-stricturing, non-penetrating (B1), stricturing (B2), or penetrating (B3) phenotypes, with or without perianal involvement. Disease location for CD was classified as ileal (L1), colonic (L2), ileocolonic (L3), or upper gastrointestinal tract (L4). For UC, we classified patients as having proctitis (E1), left-sided colitis (E2), or extensive colitis (E3).

To assess clinical disease activity in CD, we used the Harvey-Bradshaw Index (HBI)^11^, and for UC, we used the total or partial Mayo Score^12^. The HBI evaluates parameters such as general well-being, abdominal pain, presence of an abdominal mass, number of liquid stools per day, and associated complications (e.g., joint, ocular, skin, or perianal involvement)^11^. The total Mayo Score assesses clinical aspects such as stool frequency, rectal bleeding, physician’s global assessment of disease activity, and endoscopic findings (e.g., erythema, vascular pattern loss, friability, erosions, spontaneous bleeding, or ulcerations). When colonoscopy was not available, we used the partial Mayo Score, which excludes endoscopic data^12^.

To assess disease activity, we retrospectively analyzed each patient’s electronic records from outpatient visits or hospitalizations between 2019 and 2023. Any documented disease activity during a given year was considered as the patient having active disease in that year, regardless of other assessments during the same period. Disease activity was defined as HBI ≥5 for CD. For UC, total Mayo Score ≥3 or a partial Mayo Score, ≥2 indicated active disease. Obesity was defined according to the World Health Organization (WHO) criteria, based on body mass index (BMI), calculated using height and weight (BMI=weight (kg) / height (m)²). Obesity was defined as BMI ≥30 kg/m². The normal weight (eutrophic) range was defined as BMI between 18.5 and 24.9 kg/m². Individuals with a BMI between 25 and 29.9 kg/m² were classified as overweight^13^ ^,^ ^14^.

Data were compiled using a pre-established padronized questionnaire and later entered into a database table.

To analyze the data, we compared patients and IBD characteristics as well as the IBD-targeted treatment and clinical course of IBD between obese and non-obese patients, i.e., those who were overweight or eutrophic. Continuous variables were described using mean, median, and standard deviation, while categorical variables were presented as frequencies and percentages. Comparisons of continuous variables were performed using the Student’s t-test, while categorical variables were compared using the chi-square test or Fisher’s exact test. A significance level of 5% was adopted for all statistical tests. Inferential and modeling analyses were conducted using the Jamovi software, version 2.3.

## RESULTS

A total of 447 patients were evaluated, of whom 43 were excluded. The main reasons for exclusion were incomplete data recording (n=28), pregnancy^12^ and malnutrition (n=3). Among the 404 patients included, the majority were female (61.1%) and of white race (77.5%), with a mean age of 50.09±13.64 years. The mean BMI of the study population was 27.56±5.4 kg/m², with 28.2% classified as obese. Comorbidities such as hypertension, diabetes mellitus, and dyslipidemia were observed in 74 (18.3%), 97 (24%), and 80 (19.8%) patients, respectively. Former or active smokers was identified in 33% of patients.

Regarding IBD characteristics, the mean age at diagnosis was 37.79±13.44 years, with a mean disease duration of 12.50±8.3 years. Most patients had CD (68.3%), predominantly with ileocolonic involvement (51.4%) and a non-stricturing, non-penetrating phenotype (42.4%). Perianal disease was observed in 26.8% of CD patients. Obese patients were predominantly women, and had a higher frequency of colonic CD and non-stricturing, non-penetrating behavior compared to non-obese patients (Table 1) Among the 128 patients with UC, extensive colitis was found in the majority of cases (51.6%). Sociodemographic and clinical characteristics of the study population are detailed in Table 1.

**TABLE 1:** Sociodemographic and clinical characteristics of obese and Non-Obese IBD patients.

	Total (n=404)	Non-obese (n=290)	Obese (n=114)	*P* **-value**
Gender n (%)				**0.001**
Female	247 (61.1)	163 (56.2)	84 (73.7)	
Male	157 (38.9)	127 (43.8)	30 (26.3)	
White n (%)	313 (77.5)	235 (81.0)	78 (68.4)	**0.006**
BMI (kg/m²), mean ± SD	27.56±5.4			0.841
Former or current smokers n (%)	136(33.7)	90(31.0)	46(40.4)	0.075
Age (years), mean ± SD	50.09±13.64	49.58±14.21	51.38±12.02	0.232
Age at diagnosis (years), mean ± SD	37.79±13.44	37.26±13.58	39.14±13.02	0.207
IBD duration (years), mean ± SD	12.50±8.3	12.52±8.44	12.44±7.98	0.931
Type of IBD n (%)				0.977
CD	276 (68.3)	198 (68.3)	78 (68.4)	
UC	128 (31.7)	92 (31.7)	36 (31.6)	
CD Location n (%)				**0.022**
L1	72 (26.1)	57 (28.8)	15 (19.2)	
L2	51 (18.5)	31 (15.7)	20 (25.6)	
L3	142 (51.4)	99 (50.0)	43 (55.1)	
L4	11 (4.0)	11 (5.6)	0 (0.0)	
CD Phenotype n (%)				**0.030**
B1	117 (42.4)	78 (39.4)	39 (50.0)	
B2	101 (36.6)	82 (41.4)	19 (24.4)	
B3	58 (21.0)	38 (19.2)	20 (25.6)	
Perianal CD n (%)	74 (26.8)	56 (28.3)	18 (23.1)	0.379
UC Location n (%)				0.161
Left-sided colitis / proctitis	62 (48.4)	41 (44.6)	21 (58.3)	
Extensive colitis	66 (51.6)	51 (55.4)	15 (41.7)	
EIM n (%)	113 (28.0)	80 (27.6)	33 (28.9)	0.784

¹ BMI: body mass index. IBD: inflammatory bowel disease. SD: standard deviation. CD: crohn’s disease. UC: ulcerative colitis. L1: Ileal. L2: Colonic. L3: Ileocolonic. L4: Upper gastrointestinal tract. B1: Non-stricturing, non-penetrating. B2: Stricturing. B3: penetrating. EIM: extraintestinal manifestation.

Biologic therapy was used in most patients (58.4%) during the time period evaluated and treatment failure occurred in 56.8% of these individuals. During the same period, 36 patients (8.9%) required surgical treatment due to IBD-related abdominal complications, and 9 of them developed postoperative surgical complications. Additionally, 23 patients (5.7%) experienced severe or opportunistic infections. 

Table 2 depicts the IBD-targeted treatment and disease course according to the presence or absence of obesity. No statistically significant differences were observed in most of the outcomes analyzed, except for a higher frequency of biologic therapy use in non-obese patients with CD (*P*=0.028).

**TABLE 2:** Treatment and outcomes of IBD patients, obese and non-obese, from 2019 to 2023.

	Total (n=404)	Non-obese (n=290)	Obese (n=114)	*P*
Use of biologics n (%)	236 (58.4)	177 (61.0)	59 (51.8)	0.089
CD	193 (69.9)	146 (73.7)	47 (60.3)	**0.028**
UC	43 (33.6)	31 (33.7)	47 (60.3)	0.969
Failure of biologics n (%) +	138 (56.8)	99 (54.4)	39 (63.9)	0.193
CD	115 (57.8)	82 (54.7)	33 (67.3)	0.119
UC	23 (52.3)	82 (54.7)	6 (50.0)	0.853
Surgery n (%) ++	36 (9.0)	27 (9.3)	9 (7.9)	0.646
CD	31 (11.3)			
UC	5 (3.9)			
Surgical complication n (%)	9 (25.0)	6 (22.2)	3 (33.3)	0.730
CD	8 (25.8)			
UC	1 (20.0)			
Severe or opportunistic infection n (%)	23 (5.7)	18 (6.2)	5 (4.4)	0.477
Hospitalizations (n) mean ± SD	0.33±0.69	0.31±0.65	0.35±0.80	0.551

IBD: inflammatory bowel disease. SD: standard deviation. CD: crohn’s disease. UC: ulcerative colitis. **+**biologic failure includes need for dose optimization or switching of biologic class. **++**surgeries include bowel resection procedures for IBD treatment, excluding perianal surgeries.

Regarding disease activity, each patient was evaluated annually between 2019 and 2023 in each case record of your outpatient visit, and disease activity was determined for each year. Subsequently, the number of years with active disease was calculated and compared between obese and non-obese patients, separately for the CD and UC groups, and in the overall IBD population (Table 3). There was a trend toward a higher number of years with active disease among obese patients with CD (*P*=0.062).

**TABLE 3:** Mean number of years with active disease in patients with CD and UC (2019-2023).

	Total	Non-obese	Obese	P
CD (years), mean ± SD	1.29±1.57	1.16±1.43	1.60±1.86	0.062
UC (years), mean ± SD	1.47±1.36	1.51±1.49	1.36±1.12	0.578
Total (years), mean ± SD	1.34±1.51	1.27±1.44	1.53±1.66	0.148

SD: standard deviation. CD: crohn’s disease. UC: ulcerative colitis.

## DISCUSSION

The present study showed a higher prevalence of women with IBD overall, and when analyzing gender within the study groups, obesity was significantly more prevalent among women than men. In fact, in Brazil, data from the 2019 National Health Survey (PNS/2019) show that obesity is more prevalent in the female population-30.2% of women versus 22.8% of men^15^. Additionally, regarding race, there was a high prevalence of white individuals in both groups, also with statistical significance, consistent with findings in the literature^1^ ^-^ ^4^.

Historically, IBD was associated with malnutrition and underweight patients. However, recent studies^3^ ^,^ ^4^ have demonstrated increasing rates of overweight and obesity in the IBD population, similar to the general population, aligning with what we found in this study-obesity was present in 28.8% of the sample. This figure is consistent with national data. According to ABESO (Brazilian Association for the Study of Obesity and Metabolic Syndrome), in 2019, one in every four individuals aged 18 or older in Brazil was obese, totaling around 41 million people-29.5% of women and 21.8% of men^16^.

Regarding IBD subtype, most patients in our sample had CD (68.3%). However, obesity was similarly prevalent among patients with CD and UC - 28.26% and 28.12%, respectively. This finding contrasts with most studies in the literature, such as a cohort conducted in South Korea that examined waist-to-hip ratio and IBD risk in over 10 million participants followed for 9.3 years, showing that CD was associated with abdominal obesity^17^. This trend had already been observed more than a decade ago, as demonstrated in a cohort meta-analysis including over 600,000 participants, which found a strong association between BMI and CD risk^18^.

Among patients with CD, we observed a significantly higher prevalence of the non-stricturing, non-penetrating phenotype in the obese group. This finding aligns with the literature and is explained by the production of proinflammatory cytokines such as TNF-α, interleukins, and other adipocytokines secreted by adipose tissue, particularly visceral fat in obese IBD patients^19^. Regarding disease location, we found a predominance of ileocolonic involvement among obese patients. No significant difference was observed regarding the presence of perianal disease. This contrasts with most studies reporting an association between obesity and perianal involvement in CD patients, with a higher likelihood of developing perianal abscesses and complex fistulas in this population^3^ ^,^ ^4^. We speculate that genetic differences between Brazilian and European or North American populations may, at least in part, justify these phenotypic differences. On the other hand, for UC, no statistically significant difference was found regarding disease location or the presence of EIMs between obese and non-obese patients.

Some evidence in the literature suggests that obesity is associated with a more aggressive IBD course, including perianal manifestations in CD, while other studies have not confirmed this association. Several cohort studies in patients with immune-mediated inflammatory diseases (IMIDs), including IBD, treated with biologics such as anti-TNF agents, have suggested that obesity is associated with poorer response to biologic therapy^20^. In the present study, most patients (58.4%) received biologic therapy. Among them, treatment failure occurred in 56.8% during the study period (2019-2023), requiring either dose optimization or a change in therapeutic class. Overall, we did not observe statistically significant differences between obese and non-obese groups in terms of the need for or failure of biologic therapy. However, when analyzing CD patients specifically, the need for biologics was significantly higher in non-obese patients (*P*=0.028), while biologic therapy failure was more common in the obese group, though not statistically significant. These findings are consistent with some studies reporting that obesity is not necessarily associated with an increased need for biologics but, once initiated, is linked to poorer outcomes, requiring dose adjustments or medication changes^2^ ^,^ ^21^. To our knowledge, this is the first Brazilian study to evaluate the impact of obesity on the long-term clinical course of IBD.

The literature lacks a consensus on whether obesity contributes to a more aggressive IBD course requiring or resulting in biologic therapy failure. Biologic failure may be related to the altered pharmacokinetics of these drugs, with faster clearance in obese patients^19^ ^,^ ^22^, as well as obesity-related inflammation potentially enhancing disease severity. Elevated TNF levels produced by visceral fat in obese patients may lower effective trough levels of anti-TNF agents, possibly requiring higher doses for efficacy^19^ ^,^ ^21^. Other studies indicate that the impact of obesity on drug clearance varies by biologic type-greater with TNF antagonists like infliximab and adalimumab and potentially less so with vedolizumab and ustekinumab^23^. More research is needed to fully understand these mechanisms and determine whether weight loss may reduce the risk of developing IBD or improve its course in obese individuals. In the meantime, the relationship between obesity and IBD may be considered bidirectional - obesity may increase IBD risk and worsen its prognosis, while IBD may contribute to the development of metabolic disorders such as hepatic steatosis^24^. Therefore, addressing the pandemics of metabolic diseases and IBD requires lifestyle changes - adopting a healthier, fiber-rich, low-processed-food diet, increasing physical activity, improving sleep hygiene, among others-which are essential for improving health in the IBD population^3^ ^,^ ^4^.

Regarding disease activity during follow-up, we observed a trend toward higher disease activity (mean number of years with active disease) in obese patients with CD compared to non-obese patients (*P*=0.062). This trend was previously reported more than a decade ago in studies of IBD patients treated with infliximab, which showed that obese individuals had a 3- to 9-fold increased risk of disease flare and therapy optimization compared to eutrophic patients^25^. This issue has also been observed in other autoimmune diseases requiring biologics, as demonstrated in a large systematic review including 54 cohort studies with 19,372 patients with autoimmune diseases treated with anti-TNFα. In 23% of the obese population, the risk of biologic failure was 60% higher than in non-obese patients - each 1 kg/m² increase in BMI was associated with a 6.5% higher chance of treatment failure^26^. However, these findings are not uniform in the literature. For instance, in a clinical trial with 254 IBD patients using ustekinumab, no significant differences were observed in long-term endoscopic remission between obese and eutrophic patients^27^.

Conversely, there was no statistically significant difference in years with disease activity among UC patients with and without obesity. However, the literature presents conflicting findings - one cohort study with 160 UC patients on biologic therapy found that each unit increase in BMI was associated with a 4% increase in the risk of biologic failure, a 8% increased risk of IBD-related surgery or hospitalization, and a 6% decrease in the likelihood of achieving endoscopic remission^28^. In contrast, secondary analyses of a clinical trial involving UC patients treated with infliximab showed no association between obesity and poorer endoscopic response^26^.

In addition, we did not observe significant differences in the need for surgery due to IBD-related complications between obese and non-obese groups. However, it is well-established that obese patients undergoing abdominal surgeries are more prone to surgical complications^4^ ^,^ ^19^ ^,^ ^29^. Given the small number of patients requiring surgery in our cohort-most of them with CD - there may have been a statistical bias or a type II error in this analysis. Similarly, we also did not find statistically significant differences between obese and non-obese patients regarding the occurrence of severe or opportunistic infections or hospitalizations due to IBD, in both CD and UC groups. It is possible that adequate control of IBD inflammatory activity through biologic therapy in both groups contributed to similar infection rates and hospitalization frequencies. In fact, IBD activity and frequent corticosteroid use are well-known major risk factors for severe or opportunistic infections in this population^30^.

One of the limitations of our study was the single time-point assessment of obesity, which made it impossible to determine when obesity began or to assess weight variation over time. Another limitation was defining obesity solely based on BMI, without distinguishing between the presence or absence of visceral obesity. Additionally, being a retrospective study, we faced limitations due to incomplete or inaccurate records in electronic and physical medical charts. Lastly, there was a selection bias since the study was conducted at a tertiary hospital (HU-UFJF), a referral center for IBD, thus including patients with more severe disease profiles, which may limit the generalizability of our findings.

Despite the above-mentioned limitations, our study has several strengths. It highlights the impact of obesity on the clinical course of IBD, particularly in CD, in a large cohort of patients treated with various IBD-targeted therapies with follow-up for four and a half years. Moreover, the present study provided real-world evidence regarding both, the high need for biologic therapy in obese and non-obese IBD patients and the frequent need for optimization or class switching of therapies over the course of follow-up in a tertiary care center for IBD patients. Additionally, in this study, patient data extraction was obtained through a standardized electronic form, which allows for more reliable data capture.

Future long-term, preferably controlled prospective, multicenter studies involving broader obese and non obese IBD populations, aiming to explore the clinical course of IBD during treatment with various advanced IBD-targeted therapies will be very useful and may contribute to expanding knowledge regarding the impact of obesity on long-term IBD outcomes.

## CONCLUSION

Obese IBD patients were predominantly women, and had a higher frequency of colonic CD and non-stricturing, non-penetrating behavior compared to non-obese patients. Among those with CD, disease course was characterized by greater disease activity over time, suggesting that obesity may be an unfavorable factor for disease control in CD. This trend was not observed among patients with UC. Conversely, there was a greater need for the use of biologics in non-obese CD patients. Further studies, preferably prospective and controlled, are needed to better determine the impact of obesity on the course of both UC and CD.

## Data Availability

Data-available-upon-request
